# Post-esophagectomy chylothorax refractory to mass ligation of thoracic duct above diaphragm: a case report

**DOI:** 10.1186/s13019-022-02001-7

**Published:** 2022-10-06

**Authors:** Shengxi Wang, Wenpeng Jiang

**Affiliations:** 1 Department of Oncology, Shandong Provincial Hospital Affiliated to Shandong First Medical University, No. 324 Jingwu Road, Huaiyin District, Jinan, 250000 Shandong China; 2 Department of Thoracic Surgery, Shandong Provincial Hospital Affiliated to Shandong First Medical University, No. 324 Jingwu Road, Huaiyin District, Jinan, 250000 Shandong China

**Keywords:** Refractory chylothorax, Variation of lymphatic system, Thoracic duct ligation

## Abstract

**Background:**

Post-esophagectomy chylothorax is a relatively rare but potentially lethal complication. The treatment strategy of post-esophagectomy chylothorax remains a subject of debate which mainly focuses on the indication and timing of surgical intervention. For cases in which the leakage site is not localized, a mass ligation of the thoracic duct above diaphragm is advocated as the surgical procedure is believed to ensure sealing all the accessory ducts that could be the source of the chylothorax. But in this paper, we report a case of post-esophagectomy chylothorax which was refractory to mass ligation of thoracic duct above diaphragm.

**Case presentation:**

A 59-year old man suffered from high output chylothorax (> 1000 ml/24 h for more than 30 days) after esophagectomy through left thoracotomy. Considering the failure of lymphangiography, we performed mass ligation of thoracic duct above diaphragm. However, we failed to close the chylous leakage. Finally, we found that a rare variated tributary of thoracic duct was the resource of the chylous output. Both the variation of lymphatic system and the coincidence of injured site lead to the invalidness of reoperation. After definitely ligating the variated tributary, chylothorax was cured.

**Conclusion:**

This case supplies a direct evidence that mass ligation of thoracic duct is of no avail in some refractory chylothorax, which indicates the importance of chylous leakage localization.

## Background

Among traumatic chylothorax, esophagectomy is one of the most common iatrogenic causes. The incidence of chylothorax after transthoracic esophagectomy noted in the literature ranges from 0.4 to 9% [1, 2]. Chylothorax complicating with esophagectomy represents high mortality because a high daily chylous output can lead to dehydration, electrolyte abnormality, undernutrition and lymphopenia. However, the management of postoperative chylothorax remains controversial. Although kinds of lymphangiography are among the modalities for the identification of chylous leakage as well as for the minimally invasive embolization, the success rate of the procedure is uncertain due to the technical challenging and individuality [3, 4]. Surgical treatment is imperative in some cases [5]. Nevertheless, the indication, surgical approaches and timing of surgical intervention are still a debate due to the high invasion and sometime nullity [6].

In this paper, we presented a case of refractory postoperative chylothorax in which surgical intervention by ligaturing the thoracic duct above the diaphragm failed to close the leakage. To the best of our knowledge, this is the first report that a rare variation of thoracic duct and the coincidence of injured site lead to a refractory post-esophagectomy chylothorax.

## Case presentation

A 59-year old man presented with dysphagia was admitted in our department. By endoscopy, an irregular ulcerated lesion extending 31 to 35 cm from the incisal dentition was noted. Then esophageal squamous cell carcinoma was diagnosed by endoscopic biopsy and the clinical staging was identified as T3N0M0 (IIA staging) by the Union for International Cancer Control (UICC) TNM staging system [7]. After a multidisciplinary consultation including oncology, the patient underwent esophagectomy via left open thoracotomy, as there was no suspected upper mediastinal lymph node metastasis evaluated by preoperative CT scan. The surgery was taken smoothly.

On the first day after surgery, the left pleural effusion drainage was about 400 ml, with bloody appearance. After a liquid diet was given to the patient through a duodenal feeding tube, the pleural effusion drainage increased sharply to 2900 ml and the appearance changed to serosanguinous, and then milky. Laboratory tests confirmed the chylous pleural effusion (Table 1).

**Table 1:** Characteristics of pleural effusion

Pleural fluid	Value	Reference value
Protein (g/L)	20.9	10–20
LDH	310 U/L	˂50% of plasma level (100–300 U/L)
Leukocyte ( cells/mL)	3200	450
Lymphocyte (%)	67	10
Triglyceride (mmol/L)	11.3	< 1.24
Cholesterol (mmol/L)	0.28	< 5.17

**Fig. 1:**
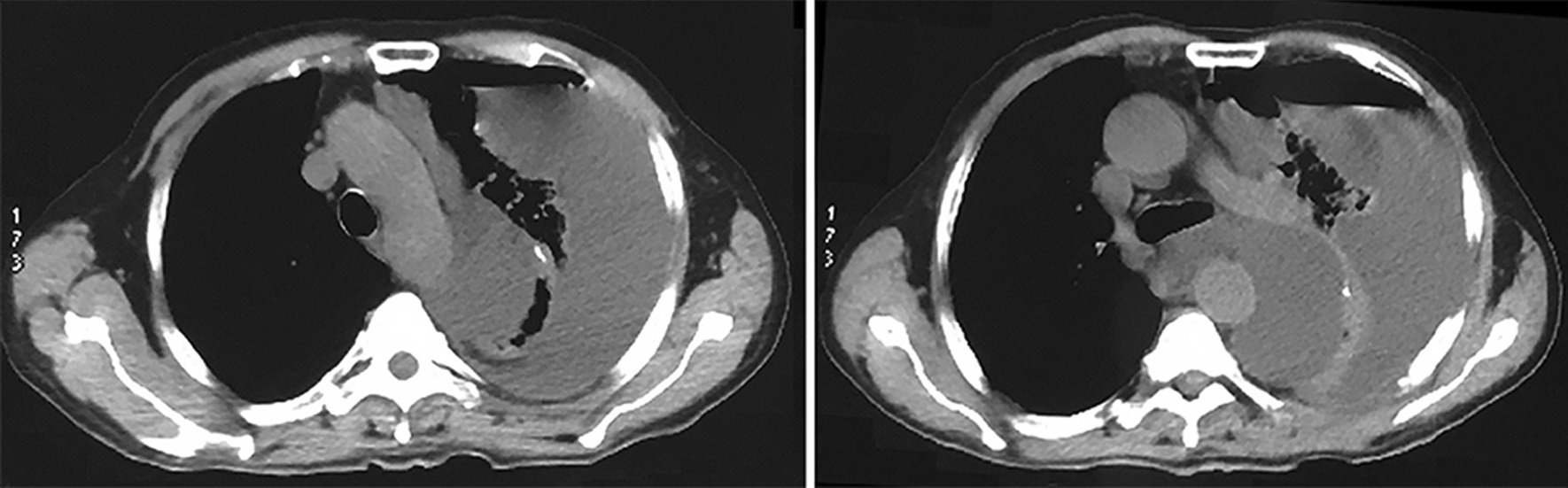
The thoracic CT scan showed a huge cyst full of fluid in the left thoracic cavity

Chylothorax was diagnosed and then total parenteral nutrition (TPN) and octreotide (300 ug/day) were employed. The drain output maintained about 1500 ml per day, with a serous gross appearance. Considering the high output of chylous leakage, reoperation was proposed, but relatives of the patient refused. Then we tried lymphangiography via a pedal approach but failed due to the puncture failure. On the 33rd day after esophagectomy, the drainage decreased sharply to 300 ml per day and the patient took a complaint of chest congestion and hiccup. A lower breath sound was revealed by chest auscultation. The Chest CT scan showed a huge cyst full of fluid in the left thoracic cavity, indicating the formation of compartments in left thorax and difficulties of adequate drainage (Fig. 1).

Reoperation was imperative. The patient received mass ligation of thoracic duct above the diaphragm. On the first day after reoperation, the chest tube drainage was about 1300 ml, with serosanguinous appearance. Then, the drainage was above 1500 mL on average in the following days (Table 2). The chylous test of discharge was positive. Unfortunately, the surgical procedure did not work.

**Table 2:** Trend of intercostal drain output and the management

Postoperative day	Left chest drain (ml)	Management
1	400	
2	300	
3	500	
4	200	
5	50	
6	50	
7	2900	Started conservative medical treatment (including TPN, octreotide and so on)
8	1500	
9	1050	
10	1300	
11	2300	Tried to perform lymphangiography, but failed
…	1100–1500	
33	350	Chest CT
34	300	The second operation (thoracic duct ligation)
1(the 2nd operation)	1300	
2	1400	
3	1150	
4	1800	
5	1500	
6	1750	
…	1700–3500	
20	3200	The third operation (found the leakage site and the tributary of thoracic duct and ligated the tributary)
1(the 3rd operation)	200	
2	140	
3	60	
4	40	Oral low-fat diet administration
5	20	
6	50	Removed the chest tube
…		
16		Discharged

**Fig. 2:**
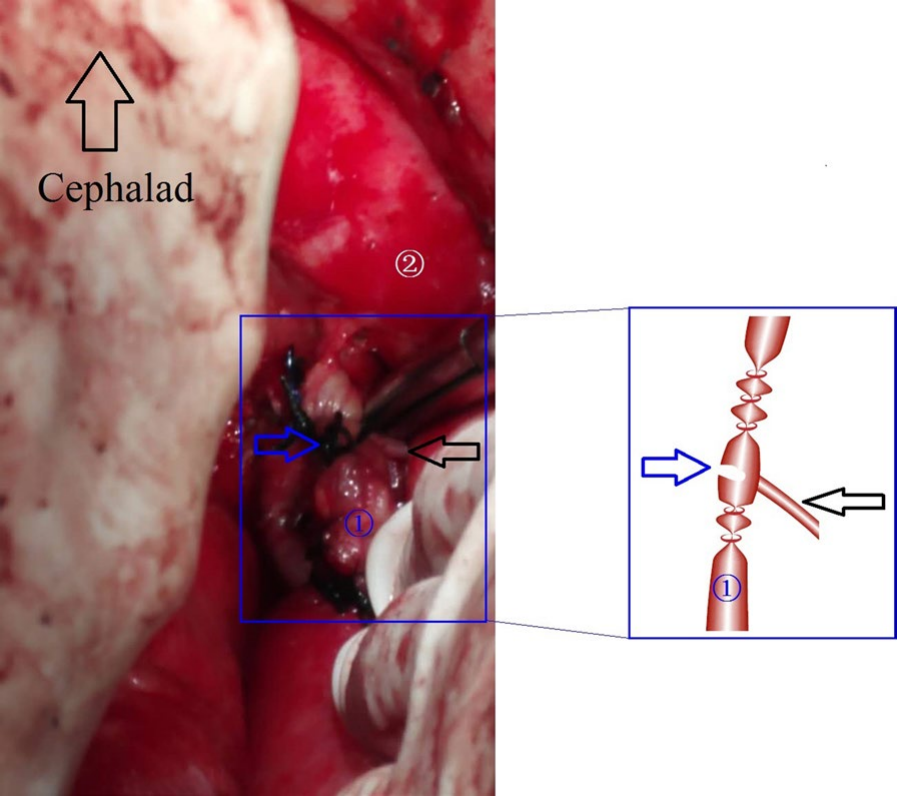
Intraoperative findings showed the variated tributary of the thoracic duct (the black arrow), the leakage of the thoracic duct (the blue arrow). ① The thoracic duct (after ligation) and ② the aortic artery

After adequate preoperative preparation, a third operation was scheduled. Six hours before surgery, olive oil was given through the duodenal feeding tube. After separating the adhesions, we found that the trunk of thoracic duct was definitely ligated. But there was still clear liquid accumulation at the lowest of the posterior mediastinum. Finally, the leakage was found in the anterior wall of the thoracic duct on the level of the carina. Then, we dissected the thoracic duct near the leakage, and found that a tributary was placed into the thoracic duct in the opposition of the leakage (Fig. 2). Lymphatic fluid drained by the tributary entered the thoracic cavity by the injury of the thoracic duct which located opposite the tributary. Finally, the chylothorax was cured after the tributary had been duly ligated.

## Discussion and conclusions

The treatment of post-esophagectomy chylothorax ranges from conservative strategy to surgery. Despite the development of lymphangiography and thoracic duct embolization, surgery remains the cornerstone in the management of chylothorax refractory to medical management [6]. Some surgeons argued that surgery should be attempted only after conservative management fails [8]. Meanwhile, some insisted that surgical intervention should be initiated if the chylous output remains > 10 ml/kg/day after 48–72 h of conservative management as the likelihood of spontaneous resolution is less [9].

Surgical thoracic duct ligation can be performed via abdominal, thoracic and cervical approaches [10]. Among the approaches many surgeons prefer a mass ligation of the duct above the esophageal hiatus between the aorta, azygous vein, vertebral bodies, and the pericardium. It is believed that this procedure ensures duct ligation at its entry in the chest, so it can seal all the accessory ducts that could be the source of the chylothorax. Therefore, mass ligation of thoracic duct was recommended in cases in which the loss of chyle is not identified [5]. In this paper, we present an intuitive and concrete situation in which mass ligation of thoracic duct above the diaphragm failed in closing chylous leakage after esophagectomy.

In this case, we did not identify the exact site of chylous leakage because of the failure of lymphangiography. So we performed the mass ligation of thoracic duct just above the diaphragm in order to close the postoperative chylous leakage, but we failed. The chylous leakage output was more than 1100 ml per day after the surgical procedure. It was a severe situation as the patient might get depleted nutritionally and immunologically. It is the proper medical therapy that the patient can undertake the reoperation without serious complications. There was no other choices but the arrangement of the third operation. Failed to identify the leakage site preoperatively, we tried our best to find it intraopertively. Olive oil was administrated through a nasogastric tube 6 h before operation, which can facilitate the visualization of the leak in the thoracic duct by increasing the flow of lymph. Finally, in the process of the third operation we clarified the reason of invalid thoracic duct ligation. We found that a variated tributary ends into the thoracic duct just opposite the injured wall of the thoracic duct on the level of the carina. Lymphatic fluid drained by the tributary entered the thoracic cavity through the injury of the thoracic duct. In conclusion, the variated tributary and the coincidence of the injured site on the thoracic duct collaborated in the invalidation of reoperative thoracic duct ligation. Meanwhile, from this case we confirmed that mass ligation of thoracic duct above the diaphragm can not guarantee definite ligation of all the branches of thoracic duct due to the variation of lymphatic system. This case supplied the direct evidence that mass ligation of thoracic duct above diaphragm is invalid in some chylothorax, which had not been reported. All efforts should be taken to identify the chylous leakage site including preoperative lymphangiography or careful observation during operations, which can help to avoid the failure of the surgical intervention.

There is one limitation in our report. We did not identify the origin of the variated tributary due to the failure of lymphangiography.

In conclusion, post-esophagectomy chylothorax must be managed promptly to avoid serious consequences. It is the key point to identify the precise location of the leakage, which can ensure the definite ligation. Adequate preoperative preparation is also crucial to the reoperation.

## Data Availability

The materials in the paper, including all raw data, will be freely accessible to any scientist who wishes to get them for noncommercial purposes, without breaching participant confidentiality.

## Abbreviation

TPN: Total Parenteral Nutrition
